# Environmental Influences on Individuals with Autistic Spectrum Disorders with Special Emphasis on Seasonality: An Overview

**DOI:** 10.3390/children10121851

**Published:** 2023-11-25

**Authors:** George Botsas, Eirini Koidou, Konstantinos Chatzinikolaou, George Grouios

**Affiliations:** 1 Department of Early Childhood and Care, School of Social Sciences, International Hellenic University, 57400 Thessaloniki, Greece; 2 Department of Education, School of Education and Social Sciences, Frederick University, 3080 Limassol, Cyprus; 3 Department of Human Performance, School of Physical Education and Sports Sciences, Aristotle University of Thessaloniki, 57001 Thessaloniki, Greece; rkoidou@phed-sr.auth.gr (E.K.); konchatzinikolaou@phed.auth.gr (K.C.); ggrouios@phed.auth.gr (G.G.)

**Keywords:** autism, seasonality, time of birth, infection, nutrition, vitamin, geographic location, photoperiod, immigration

## Abstract

This paper offers an in-depth exploration of the intricate relationship between environmental factors and autism spectrum disorder (ASD), with a special emphasis on seasonality. It reviews existing research, providing a comprehensive summary of findings and highlighting the multifaceted dimensions of several environmental factors influencing the etiology of ASD. The discussion encompasses various elements, including birth months, maternal health, dietary choices, and vitamin D deficiency, delving into the intricate interplay of seasonality with environmental influences such as viral infections and solar radiation. The present study raises essential questions regarding the timing of environmental influences and the factors contributing to the rising prevalence of ASD. Ultimately, it underscores the need for future epidemiological research to incorporate more extensive investigations of environmental risk factors and employ advanced statistical analyses. This comprehensive overview contributes to a deeper understanding of how environmental factors, particularly seasonality, may be linked to the occurrence of ASD and its increasing prevalence, recognizing the multifaceted and diverse nature of these interactions.

## 1. Introduction

Autistic spectrum disorder (ASD) is a neurodevelopmental condition characterized by the presence of consistent and often severe deficits in social communication and interaction among individuals diagnosed with it, along with challenges in adapting to changes in their environment [1]. The manifestation of this disorder is marked by notable deficits within the framework of what is known as the “triad of impairments,” encompassing social interaction, communication, and flexible imaginative abilities [2].

The most recent edition of the *DSM-V-TR* [3] outlines two fundamental diagnostic criteria. Criterion “A” encompasses deficits in both social interaction and social communication, which entail specific behaviors. Criterion “B” provides detailed descriptions of the behaviors commonly observed in individuals with ASD, such as recurrent motor stereotypes, the use of objects, echolalia, adherence to routines and rituals, and sensory abnormalities. This disorder’s onset is now recognized in the early stages of development and exerts a significant impact on an individual’s educational, social, and professional aspects of life [4].

The prevalence of ASD has been the subject of numerous early studies. For instance, a study conducted in 1966 in Middlesex, United Kingdom [5], reported that ASD affected approximately 2 individuals in every 10,000 live births. However, as subsequent years unfolded, a growing number of children were diagnosed with autism, leading to an increase in the prevalence rate to 5 in 10,000 people. One plausible explanation for this variation in prevalence rates could be the presence and quality of birth and health registries in different countries. Wherever comprehensive registers were maintained, the prevalence of autism tended to be notably higher [6].

The question that is currently bothering researchers goes beyond just the prevalence of autism. ASD is an intricate and lifelong condition with a clear neurodevelopmental basis. It is evident that the roots of ASD are embedded in a hereditary predisposition involving specific genes [7]. Research on this subject has now identified more than 100 genes associated with an increased risk of autism, further complicating our understanding of ASD [8]. What adds to the complexity is the emergence of studies emphasizing the role of environmental factors and their interaction with genes in the etiology of autism [9,10], hinting at the multifactorial nature of the disorder.

The aim of this paper is to provide a comprehensive overview of the complex relationship between seasonality and ASD. It seeks to summarize existing research, highlight the diverse dimensions of seasonality, and examine the potential influence of the complex interaction between environmental, genetic, and fetal brain development factors on the development of ASD. Overall, the present study endeavors to illuminate the multifaceted nature of seasonality and its significance within the context of autism.

## 2. Literature Search Strategy

Studies eligible for inclusion in this review included original research studies, critical reviews, literature reviews, rapid reviews, scoping reviews, umbrella reviews, systematic reviews, meta-analyses, and overviews in the English language. The studies examined the impact of environmental factors on individuals with autism spectrum disorder (ASD). The search was conducted on MEDLINE and EMBASE, Cochrane CENTRAL, ISI Web of Science, and SCOPUS. We used keywords such as autism, ASD, risk factors, environmental factors, prenatal, pregnancy, seasonality, month of birth, infection, fever, antibiotics, nutrition, obesity, BMI, vitamin, geographical location, photoperiod, immigration, pollution, toxics, and pesticides.

In contrast to primary researchers, reviewers do not collect deeply personal, sensitive, or confidential information from participants. Reviewers use publicly accessible data, documents, or records as evidence and are not required to seek institutional ethics approval before commencing a review study. Institutional Review Boards, responsible for ethical conduct in research, typically do not provide guidelines specifically for review studies. Our universities are among those institutions.

## 3. Seasonality

Seasonality pertains to the occurrence of pronounced peaks in childbirth during specific months or seasons within a year. Research has shown a correlation between the presence of these noticeable birth peaks and the incidence of ASD, while other months exhibit a more uniform distribution of births [11] (pp. 308–309).

The concept of specific timing for various events is exemplified in the Old Testament book of “Ecclesiastes”, which famously states, “To everything, there is a season, and a time to every purpose under the heaven. A time to be born, and a time to die; a time to plant, and a time to pluck up that which is planted” [12] (chapter 3, pp. 1–2). Centuries earlier, the Greek physician Hippocrates (460 BC) emphasized the importance of considering the seasons in medicine when he wrote in his work “*On Winds, Waters, and Places*”, “Whoever wants to follow the science of medicine in a direct way must first investigate the seasons of the year and what happens in them” [13].

In this context, when epidemiological studies reveal a seasonal pattern in the occurrence of a disease or disorder that deviates from a uniform distribution of births, it suggests the presence of the disorder very early in a child’s life. This, in turn, implies that season-related factors might have an impact on the etiology of the disorder [8,14]. This pattern is also observed in the case of autism.

## 4. Seasonality Dimensions

The term “seasonality” pertains to the correlation between the timing of birth and the likelihood of a disorder occurring in offspring. There are three key hypotheses related to the seasonality of human births [15] (p. 379): (a) seasonality linked to social factors affecting the frequency of sexual intercourse, (b) seasonality associated with climatic factors impacting human fertility, and (c) seasonality influenced by energy-related factors having a specific effect on female fertility. These three hypotheses have directed research towards investigating environmental risk factors related to seasonality, thus shedding light on various aspects of the etiology of autism.

The initial dimension of birth season may encompass a broad spectrum of events and circumstances that could collectively influence the mechanism underlying the development of ASD. These factors may represent either overall risk factors or individual risk factors contributing to the occurrence of ASD.

The seasonality aspect as a risk factor for autism can be linked to various elements, including viral infections that tend to be more prevalent during certain months, temperature fluctuations and climate changes specific to each season, dietary habits of pregnant women influenced by the season, deficiencies in certain health indicators like vitamin D, which is intricately tied to photoperiod, solar radiation (UVB), and consequently, the geographical location of the parents at the time of birth.

In recent years, there has been a growing focus not only on the birthplace of the child but also on the migration history of the parents as a potential risk factor for the development of ASD [16,17]. Hence, exposure, particularly of the mother, to certain environmental factors like specific seasonal dietary habits, malnutrition, viral infections, solar radiation, or climatic conditions, is linked to seasonality [18].

## 5. Time Dimension

In these studies, examining the connection between the month of birth and ASD is important to clarify that the influence is not attributed to astrology or zodiac signs. Instead, it is assumed to be associated with medical factors [19]. These investigations, which explore the month of birth or even conception as a potential risk factor for developing ASD, have produced varied results, with some demonstrating a positive correlation and others showing negative or mixed outcomes.

Starting in the 1980s, the impact of birth month on the development of ASD began to be explored [20]. In 1981, Bartlik [21] discovered a statistically significant difference in the birth rate of children with ASD for those born in March and August compared to the general population. Similarly, Konstantareas and colleagues [22] found a higher number of births of children with autism during the spring and summer, particularly in March, May, and June, with a lower number found during the autumn and winter seasons. This trend continued with findings showing a link between summer birth and ASD [23].

A study by Mouridsen and his team [24] involving children with Intellectual and Developmental Disabilities (IMPs) born from 1945 to 1980 revealed higher birth rates for boys with autism in March during the periods of 1951–1956, 1963–1968, and 1975–1980. However, no consistent peaks in ASD births were found throughout the entire study period.

The influence of birth season on the prevalence of autism has been observed in various studies conducted in different regions. These include spring effects, especially in March, for low-functioning individuals [25], March–April [26], and March, May, and September [27]. Other researchers identified significant risk periods for ASD births in the months of October–March [28], December–February [29], and summer in the UK [30], with a specific peak in August in Israel [31].

The seasonality-as-a-risk-factor concept has also been supported by studies in Egypt, which found higher rates in spring and winter [32]. These findings were corroborated by other researchers in Egypt, who also introduced additional factors like maternal age and perinatal health and care [33]. Similarly, a study in Syria confirmed these findings while considering additional factors such as parental kinship, their age, and smoking habits [34]. An intriguing conclusion regarding the increased risk of ASD emerged from a study on planned pregnancies in Ukraine, which factored in geographical location and the prevailing climate [35].

A notable aspect within the body of research concerning seasonality in autism is the meta-analysis of studies, exemplified by the work conducted by Gardener and his colleagues [36]. In this study, they underscored the significance of a child being born during the summer months as one of several potential risk factors associated with the onset of ASD. Researchers contend that a single environmental factor is unlikely to be the exclusive cause; instead, it is the interplay of various factors, in conjunction with seasonality, that points to maternal exposure to health-compromising conditions during pregnancy [36].

Nonetheless, it is important to note that the value of seasonality as a robust environmental risk factor for ASD is a subject of ongoing debate. For instance, Mazumdar and colleagues [37] identified a recurring but gradually decreasing pattern in the seasonality of ASD child conceptions, particularly in November. This suggests that seasonality-related environmental factors may persist for a specific and limited number of years [37] (pp. e41265).

Despite some studies demonstrating a correlation between birth month or season and the risk of autism development, there are others that have failed to provide substantial support for such relationships. Bolton et al. [38], for instance, explored various seasonality models but were unable to establish a definitive link. Similarly, [39], even after subdividing their sample of ASD children into “verbal” and “non-verbal” groups, did not observe a connection with seasonality (specifically, in March and August), and, as a result, described seasonality as a questionable risk factor for the emergence of autism [39].

Several other studies have also examined specific groups of children with ASD based on factors such as gender and intelligence [40,41,42]. Nevertheless, these studies did not substantiate the hypothesis of a seasonality effect. However, it is worth noting that even researchers who did not find sufficient evidence to support the hypothesis acknowledged the possibility of a seasonal variation in the birth of a child with ASD [43,44].

This possibility has led researchers to formulate the hypothesis that the role of seasonality is far more intricate than simply identifying one or more months in which the births of children with ASD surpass the uniform distribution of other months. Many studies explore the effect of birth month in conjunction with various other factors or dimensions of seasonality as significant risk variables for the onset of autism. The amalgamation of factors, including maternal exposure to viral infections during pregnancy, dietary habits, vitamin D deficiency, the geographic birthplace of the parents in connection with solar radiation levels, and migration patterns, when correlated with the birth month, is a recurring theme in several studies supporting the substantial influence of complex seasonal factors as risk factors for the development of ASD [38,45].

## 6. Viral Infection—Fever, and Medications

A woman’s pregnancy necessitates maintaining a satisfactory state of health due to the demanding nature of the process [46]. The first trimester of pregnancy is a critical period during which fetal brain development takes place, making it particularly susceptible to harm [47]. However, it is essential to note that this period alone is not a sufficient or necessary condition for the onset of a disorder.

Certain prenatal viral infections, especially those prevalent during the winter months, can introduce additional contributing factors that increase the risk of autism [47,48,49,50].

Significant peaks in the seasonality of ASD onset during the second trimester, along with perinatal infections like pneumonia and bronchiolitis, have been observed [23]. Moreover, a higher number of births of children with Asperger syndrome in March has been linked to early seasonal viral infections occurring between January and March [51,52]. The prevalence of ASD births with comorbidities, such as epilepsy, has been identified in Israel in association with meningitis virus [53,54].

Furthermore, several infections have been associated with an increased number of children born with ASD and lower functionality during the spring and summer months. These infections include rubella, herpes simplex, cytomegalovirus [55], and human immunodeficiency virus, as well as syphilis, toxoplasmosis, and varicella, all being attributed as seasonal variables contributing to the higher occurrence of ASD births in spring and summer [56].

Maternal and paternal autoimmune disorders have also been linked to ASD and are considered potential causative factors [57]. However, it is important to note that not all research groups are in agreement with this finding [58,59].

The idea of viral infections cooperating with the birth month of children with ASD has not gained unanimous acceptance. The role of a suboptimal intrauterine health environment in the development of ASD, particularly without intellectual disability, has been found to have limited significance [60]. For instance, a study investigating the impact of influenza epidemics on the risk of ASD in England did not reveal a significant relationship [61], and the same held true for Norway [62]. Neither influenza A nor influenza B viruses were associated with an increased risk of ASD development. Researchers hypothesized that any risk of autism might result from the activation of the pregnant woman’s immune system rather than direct infection of the fetus, thus influencing the development of the fetal brain [62].

Furthermore, there are no data establishing a direct link between a pregnant mother’s influenza infection and the risk of her child being born with ASD. However, a meta-analysis by Jiang et al. [63] found that overall maternal infections requiring hospitalization were associated with this risk. The connection, instead, has been identified between maternal fever and an increased risk [64,65], particularly after the 2nd [66,67] or 3rd week of pregnancy [68]. Meta-analyses of 10 studies [69] and 9 studies [70] have confirmed these findings, irrespective of the specific maternal infection.

Interestingly, a reduction in this risk was observed among mothers who reported taking antipyretic medications, such as acetaminophen (paracetamol), during pregnancy [64,67,71], although such use might increase the risk of higher fevers and, consequently, ASD in other cases [72].

The absence of a general correlation between maternal infections and the occurrence of ASD was affirmed by the research team of Zerbo and their colleagues [73]. However, correlations were identified with bacterial and viral infections. The case of the rubella virus is unique. Although the association between rubella and the onset of ASD was clinically substantiated, a rubella epidemic took place in the late 1960s, subsiding after 1972 with the introduction of the vaccine. However, even as the strength of this connection diminished, it remained a topic of interest for researchers [49]. Similar findings apply to the measles virus [74].

A comprehensive understanding of the link between viral infections in pregnant women and the occurrence of ASD in their offspring can be derived from a meta-analysis of multiple studies [75]. This analysis of 32 studies underlines the presence of a relationship that requires further elucidation and strengthening. Given the existence of methods to treat and prevent these infections, the authors suggest that it is feasible to reduce the incidence of ASD attributed to these seasonal viral infection factors [75,76].

As a result of research into the potential impact of infections on the risk of ASD, studies have been conducted on maternal immune activation by antibiotics. Nitschke et al. [77] noted that “prenatal antibiotic exposure induces changes in the maternal microbiome, which could influence the development of the infant’s microbiome–gut–brain axis” (p. 516). This, in turn, could impact the risk of neurodevelopmental disorders in the fetus, such as autism. Studies on the use of antibiotics by women during pregnancy found either small and/or non-significant associations with the risk of giving birth to a child with ASD [78,79,80].

Although these findings “should not influence clinical decisions regarding antibiotic use during pregnancy” [77] (p. 517), more and longer-duration studies need to be conducted on maternal exposure to antibiotics, both generally and specifically on certain types [81].

Furthermore, studies investigating the impact of prenatal medicine use on the risk of ASD have been conducted, with findings being somewhat mixed. In examining maternal use of antidepressants and the risk of ASD, most studies did not find a significant increase [82,83,84,85,86]. Due to these findings, antidepressant treatment should not be withheld from pregnant women with mental health concerns [86]. On the contrary, some studies found a modest increase in the risk of ASD when pregnant women used antidepressants like selective serotonin reuptake inhibitors (SSRIs) in the second and/or third trimester [87,88,89]. However, studies that reconsidered this association between maternal use of antidepressants and the risk of ASD suggested that these correlations “are likely to represent a false-positive finding” [90].

The use of antiseizure medications like lamotrigine or carbamazepine by pregnant women was found to have a rather weak or non-significant correlation with ASD offspring birth [91]. In contrast, the use of valproic acid, a medication used to treat epilepsy and bipolar disorder, was found to increase the risk of ASD [91,92].

The association of antidepressants or antiseizure medications in women during pregnancy does not seem clear or strong. Field studies need to be more thorough because determining whether pregnant women should take supplements for mental health reasons is an important question.

## 7. Nutrition—Obesity, Diabetes, BMI, and Vitamins

The timing of a child’s birth, and consequently, the timing of pregnancy, is intertwined with the dietary choices of pregnant women and their intake of essential vitamins required for a successful pregnancy. Moreover, higher consumption of specific seasonal foods by pregnant women has been associated with a reduced risk of ASD development. For instance, the consumption of omega-3 and phytate levels [93,94,95,96] has been linked to a lower risk of ASD births.

Maternal high levels of weight have been associated with metabolic disturbances. Obesity before pregnancy or weight gain in the genital area has been considered a risk factor for some neurodevelopmental disorders in offspring [97]. Numerous studies have investigated maternal obesity as an increased risk factor for giving birth to a child with ASD [97,98]. Moreover, pregnant women with obesity, as indicated by an excessive Body Mass Index (BMI), are poised to have a greater risk for ASD in their offspring [99]. Specifically, gestational weight gain has been associated with the risk of ASD, especially in male children [100,101,102].

It is commonplace in several societies to constantly monitor the health progress of pregnant women, especially regarding their weight. Therefore, understanding the connection between obesity, BMI, and the risk factors of ASD can provide important insights for monitoring weight data, particularly for pregnant women at risk [102].

Obesity has been linked to the onset of diabetes. Furthermore, maternal diabetes (type 1 or 2), whether preexisting or occurring during pregnancy, has been associated with health problems and neurodevelopmental disorders, such as ASD. Considering that diabetes affects 15% of pregnant women with an increasing trend [103] (about 7.5% as pre-existing type 1 diabetes and 5% as pre-existing type 2 diabetes) [104], approximately 88% of maternal diabetes cases are due to gestational diabetes (GDM) [105]. This makes the need for studying the combined association of obesity and diabetes with ASD risk apparent.

These two health conditions have been correlated with ASD risk in several studies [106,107,108]. However, when diabetes was examined by studies as a separate factor in the occurrence of ASD in offspring, the findings were mixed, and the conclusions complex. Cordero et al. [109] found non-significant differences in the risk for ASD compared to other developmental disorders when the mother had diabetes. This goes to show that the relationship between maternal diabetes and ASD is not specific or clear (p. 974). However, other studies supported this relationship [110,111]. In fact, Xiang et al. [112], while not finding an association with pre-existing type 2 diabetes, found an association of gestational diabetes mellitus diagnosed at 26 weeks with the risk of ASD. Guo et al. [113] confirmed this relationship between diabetes and ASD, particularly when mothers were exposed to antidiabetic treatment.

Consequently, maternal vitamin D deficiency may serve as an environmental factor that heightens the risk of ASD development in offspring by influencing fetal brain development [114,115,116,117,118]. It can also play a role in the disruption of fetal nervous system development and function [119,120].

Maternal vitamin D deficiency has been linked to various medical conditions and factors related to sunlight exposure [121,122], as well as maternal lifestyle choices such as avoiding sun exposure and adhering to medical advice [123]. An encouraging discovery from several studies is that, irrespective of its connection to the increased prevalence of autism, vitamin D deficiency can be mitigated through the administration of small vitamin supplements [124,125].

The existence of studies that do not establish a direct link between vitamin D deficiency during pregnancy and the occurrence of ASD can be attributed to the researchers’ perspective that vitamin D deficiency [126] is merely one component of a broader and more intricate environmental seasonality factor [127,128]. This factor is characterized by exposure to solar radiation UVB, which, in turn, interacts with the geographical birthplaces of both the father and mother [122,129,130] or the overall state of the pregnant woman’s immune system [131].

## 8. Geographic Location–Photoperiod–Air and Ground Pollution–Immigration

The studies discussed in the section on vitamin D deficiency raise the issue of solar radiation exposure for pregnant women. This intricate and multifaceted factor, which is associated with the birth month and specific latitude reflecting the birthplaces of the parents, may be linked to a heightened likelihood of giving birth to a child with ASD. Consequently, it has been postulated that exposure to sunlight during the first trimester of pregnancy (conception in December to February and birth in the summer, especially in July) could serve as a risk factor for ASD [38,131,132,133,134].

The geographical variation in the birthplaces of parents appears to have an impact on the prevalence of autism spectrum disorder (ASD) and Intellectual and Developmental Disabilities (IDDs) [135]. The findings indicate a decreasing risk of ASD in countries near the equator, with this trend increasing as one moves farther from the equator [136]. Indeed, the consideration of seasonality with only the child’s birth month as a factor influencing the occurrence of ASD has become less effective, with studies starting to incorporate parameters related to solar radiation and other geomagnetic factors [131,137].

Another spatial dimension at birth that appears to increase the risk of developing ASD is residing in highly urbanized areas or relocating to such areas. Researchers [138] explain that the heightened risk is more closely associated with pollution and mobility rather than the geographical location itself. Exposure to air-polluted environments, such as near-roadway sites [139,140,141], is suggested to interact with genetic variables, increasing the risk of ASD. Although the greenspace exposure of pregnant women was related to a decreased risk of autism in offspring [142], it was the high concentration of pesticides in those places that was found to increase the risk of ASD among births by women exposed to these pesticides [143,144,145,146]. For other researchers, the correlation of pesticide exposure with ASD has a synergistic impact with other maternal factors, such as “lifestyle, socioeconomic or educational status, as well as ethnicity or gender” [147] (p. 1).

On the other hand, living in industrial areas exposes pregnant women to various toxins suspected to increase the risk of giving birth to a child with ASD, especially lead [148,149,150]. However, mixture analyses of toxins did not find an increased risk for ASD [151].

Most studies commonly claim that more research in the field needs to be conducted, as “understanding how environmental chemical exposures influence DNA methylation and how these epigenetic changes modulate the risk and/or severity of ASD will not only provide mechanistic insight regarding gene-environment interactions of relevance to ASD but may also suggest potential intervention strategies for these and potentially other neurodevelopmental disorders” [152], (p. 1) [153].

In the context of environmental factors impacting the gene–environment association in ASD risk, there is a body of research concerning maternal stressful events. Stressful life events during pregnancy, such as depression or divorce, have been found to increase the risk of ASD births [154,155,156]. Special attention has been given to the association between maternal exposure to childhood abuse and the risk of autism in the subsequent generation [157]. Moreover, prenatal maternal life stress events caused by physical disasters like storms, floods, and earthquakes were related to a small-to-moderate ASD risk [154,158,159,160].

In recent years, some studies integrated into the spatial variance dimension have discovered an increase in the occurrence of ASD when parents immigrate, with a particular emphasis on their ethnicity and race, especially when originating from developing countries. A Dutch study, for instance, revealed data showing a low risk of high-functioning autism in immigrant children from developing countries but an increase in cases of immigrant children with low-functioning autism [161]. These findings were consistent with studies conducted in Sweden [162,163] and Finland for immigrant parents from Sub-Saharan Africa [17]. Conversely, studies in Scandinavia have highlighted higher rates of ASD in children of immigrant parents from Vietnam, former Yugoslavia, the former Soviet Union, and Somalia [164,165]. Overall, a residential move experienced as a prenatal maternal stressful life event has been found to increase the risk of giving birth to a child with ASD [154].

In general, there is a consistent trend of data indicating an increased risk of giving birth to a child with ASD among women who are immigrants from outside Europe and North America [166,167,168]. This trend is observed in the United Kingdom [16] and the USA for mothers of Spanish, Filipino, and Vietnamese descent [169,170], as well as in meta-analyses [171,172]. Essentially, the findings that distinguish the risk of ASD in children of immigrant parents pertain to maternal vitamin D deficiency in migrants giving birth to children with this disorder [173].

## 9. What Accounts for These Varying Findings?

ASD is a neurodevelopmental disorder with a genetic etiology, although the precise genes involved have not yet been definitively identified. What is clear is that this genetic basis is influenced by environmental factors, including seasonality.

As indicated by the brief overview of studies discussing seasonality and its dimensions in relation to autism, there is no conclusive evidence. Findings from different studies can vary, with some showing a strong correlation, others suggesting no association, and many yielding mixed results. The interaction between genetic causative factors and environmental seasonality factors is highly complex [7], and often the correlation appears to be weak.

The conflicting findings in the context of panspermia, a fundamental aspect of the differences in the autism spectrum disorder (ASD) phenotype, can be attributed to several factors. One key factor is the variation in the quality of diagnoses, even though the characteristics of autism have been clearly outlined in manuals such as the *DSM-V-TR* [3] and ICD 10 [174]. Furthermore, many of these studies tend to overlook the influence of seasonality and the prevalence of ASD in relation to comorbidity with other disorders. They often draw their samples from specific sets and groups, which can introduce research bias [175]. For instance, when the sample includes 3-year-olds, the ASD prevalence rates may appear lower due to diagnoses at older ages [96].

Many studies investigating the connection between seasonality and Intellectual and Developmental Disabilities (IMD) rely on registry data, which may have limitations as individuals within the group may lack specific information regarding individual biological factors (e.g., genetic information, prenatal viral infections, actual sunshine exposure). Consequently, this limitation restricts the scope of the study’s inquiries and the precision of its findings [6]. Since the study of seasonality dimensions demands particular measurement requirements, the methods, timing, dosage, and validity of exposure for all subjects (e.g., exposure to sunlight) can vary [175]. This variability can lead to an ecological fallacy when attempting to obtain exposure data at the group level.

Critiques of seasonal studies on the prevalence of ASD also encompass the analytical methods employed. Non-parametric analyses, like chi-square tests [39], were not originally intended for use with birth data because they do not consider the “underlying time variable continuity” [11] (pp. 308–309). The utilization of the Kolmogorov–Smirnov index, a cyclic statistical approach, may offer potential solutions to this issue.

## 10. Conclusions and Final Remarks

The theories concerning the etiology of ASD must consider the genetic underpinnings of the disorder when attempting to explain its prevalence and, most notably, its increase in recent years. It is also apparent from the multitude of studies that this is influenced by the interaction between genes and certain environmental factors, one of which is seasonality.

The approach to seasonality, simply considering the birth month of children as the sole factor influencing the risk of developing ASD, has yielded unclear and often conflicting research results. Seasonality, as a factor, is a complex and intricate entity. The timing of a child’s conception within a year is linked to several other environmental variables or dimensions that can interact with genetic factors, leading to the highest risk for ASD. These dimensions include the risk of severe viral infections during the first trimester of pregnancy. If pregnancy occurs in the first trimester during the winter months when these infections are more prevalent, one can easily discern the relationship between seasonality and the risk of developing ASD.

Furthermore, the specific month of conception during a year also implies different dietary choices for the pregnant woman. Nutrient-rich diets can provide essential substances like folic acid and vitamin D, while malnutrition or improper dietary choices can increase the risk of ASD-related challenges. This aspect of the pregnant woman’s diet is influenced by seasonality and its impact on the availability of proper nutrition.

Vitamin D, in particular, plays a significant role in fetal brain development and is closely associated with seasonality through sunlight exposure. This association is linked not only to the specific month (photoperiod) but also to the latitude of the birthplace of the child’s parents. Regions with longer photoperiods, closer to the equator, tend to have a lower risk of ASD, although this is not the sole determinant of the disorder’s prevalence. Furthermore, when delving deeper into the factor of the parents’ birthplace, the influence of maternal and paternal immigration becomes apparent, either increasing or decreasing the risk of ASD.

Within the scope of investigating seasonality concerning ASD, one objective is to enhance hygiene conditions and access to health services for pregnant women. This, to the extent possible, is related to various dimensions of seasonality and aims to reduce the risk of the disorder [176]. Additionally, broadening the perspective on the factor of maternal hygiene conditions reveals that living in air-polluted, near-road, or industrial areas, where pregnant women could be exposed to various toxins, may increase the risk of ASD. Although these findings might suggest that prenatal greenspace exposure is associated with reduced odds of ASD [142], there have been studies questioning this result due to concerns about the effect of pesticides in such sites [147].

Expanding further on the factor of mothers’ hygiene and well-being, there are findings that suggest the impact of prenatal maternal stressful life events, such as depression, divorce, abuse, or immigration, is related to an increased ASD risk [154]. Moreover, larger-scale stressful events such as storms and earthquakes, leading to stress or a post-traumatic condition, can also increase the risk [158,160].

Future epidemiological studies should prioritize the thorough processing and utilization of population-based data for autism research. These studies need to focus on investigating risk factors that encompass not just genetic information but also environmental exposures. It is essential to consider causative heterogeneity data, given the diversity of phenotypes within the autism spectrum, as well as to employ more sophisticated statistical analyses suitable for the intricate nature of seasonality’s environmental factors. These research endeavors continually raise questions aimed at clarifying the actual relationship between seasonality and the occurrence of ASD. For example, determining whether seasonality’s impact is rooted in the conception timing or the health status of the pregnant woman during childbirth is a significant query [177]. An important finding is the lack of observed seasonality effects on unplanned pregnancies, shedding light on the influence of environmental factors on the disorder’s existence and prompting further investigation [178].

Another pressing question to address is the root causes of the increased prevalence of ASD, particularly concerning the environmental factors identified through the study of seasonality. Researchers suggest that this increase may be attributed to the broadening of the autism concept, whether through redefinitions or diagnostic criteria and tools, as well as increased awareness of the disorder [179,180]. However, it is yet to be determined whether seasonality and its various dimensions contribute to this rise in ASD cases, as this would also imply a potential shift in their significance within the disorder’s etiology.

Overall, while the interaction between seasonality and ASD is far from straightforward, it highlights the need for more comprehensive and sophisticated research to uncover the intricate web of factors influencing the prevalence of this complex neurodevelopmental disorder. Understanding the role of seasonality and its dimensions may offer insights into how to mitigate the risk of ASD and potentially reduce its prevalence.

## Data Availability

Not applicable.
